# Some Ludicrous Aspects of Human Misery

**Published:** 1891-09-26

**Authors:** 


### Some Ludicrous Aspects of Human Misery.

Many persons are still travelling, or away on their holidays, more are at home and at work again. It may, therefore, prove of interest to write down some of the ludicrous aspects of human enjoyment and misery as illustrated by travel. Of course, the selfishness of human nature finds ample scope for exhibition when the human animal is moving about, away from the wholesome restraints of friends and home influences, because, in such circumstances, the natural man or woman is apt to throw off all, or nearly all, restraint, and to indulge in the pleasures to be derived from giving way to unrestrained selfishness. In a railway carriage there is a limited space it is true, but for this very reason the thoughtful and kindly can do much to add to the comfort of their companions; On the other hand, a selfish disregard for the just susceptibilities of one's fellow makes one's presence a veritable blister and discomfort. In ocean travel this holds good to even a greater extent, and if the comparatively well give a little time and thought to the sufferings and misery of the sick, the latter will be helped materially, and will cheerfully bear their troubles, whilst the helpers, by finding a pleasant occupation, may soon become proof against all the horrors of *mal de mer*. It is ludicrous to see the envy of the sick as they watch the convalescent strutting along the deck of a steamer and equally funny to note "the strut" aforesaid, accompanied, as it is usually, by a bearing which would become no one so well as the Czar of all the Russias. Now the strutter would be much happier if he helped the envious sick, and as we have hinted, he might thus learn the best remedy for sea-sickness. We have said enough, however, by way of generalisation, and must now settle down to facts.

### Doctors and Sea Voyages for Health.

Doctors have found a sea voyage [to be a very convenient prescription in many cases. If a patient gives them an infinity of trouble, if he will not follow instructions, or refrain from this or that practice, if he develops symptoms of hypochondria, or affects the morphia habit, if he is engaged in the conduct of a weary business which makes undue calls upon his energy, if he is inclined to eat or drink too much; then, and in all these cases, and many others, to order a patient a sea voyage is to render a service to one, or possibly to two people. The patient may thus obtain exactly what he wants to effect a cure, and the doctor will certainly be rid of a troublesome case, not likely to do him credit, but quite the contrary, unless some such radical methods be resorted to. We are often amused at the advice given to troublesome patients, but the advice is as nothing compared with the prescriptions handed to them for use on a voyage. One is told to take this or that specific, and another to avoid all such poisonous follies. This man may eat abundance of meat providing it is "rare" on the principle that the more underdone it is the better antidote it will prove to seasickness. Raw meat is never very appetising, but it is probably prescribed for a sea voyage on the principle that the less meat is eaten the better it will be for the squeamish voyager. Champagne is another precautionary measure popular, and naturally popular with persons of means. We have seen a rich father dosing his little girls with champagne in the discharge of what he felt to be a stern and expensive duty. These and a hundred other so-called specifics may be seen in use in any large ocean-going steamer, but the funniest, and on the whole the most enemetic under all the circumstances, is the sailors' belief in cheese. You may find the distressful tar sitting forlorn and ill in a corner by himself nibbling cheese as a sacred duty. Indeed, the worse it makes him the more he sticks to it, and the more anxiety he displays that you should nibble too. A divine famous for his sober life and judgment when at home, whom we once travelled with, believed in singing a few bars of his favourite waltz (Sweethearts) before getting up in a morning as a specific for sea sickness. If the sea was very rough and he felt ailing he would repeat it more than once in the course of the day. In this way he kept up his spirits, cultivated a feeling of cheerfulness, and thus proved that merriment has a new use very little known to the majority of mankind, for our parson thrived on his waltz, and well too.

No wonder then that doctors content themselves for the most part with prescribing a sea voyage, and that the wisest physician is he who adds merely a small bottle of Epsom salts to be taken in water *hot*.

### The Start.

At few periods of the journey do the ludicrous aspects of human misery display themselves to better advantage than at the start. Anyone who will go to one of the large metropolitan termini and see off a tidal express may witness some of them readily enough. There is a class of people who actually become green, yellow, and all the other colours induced by sea voyaging, as the train prepares to start from the port of departure. We always associate toilet vinegar and other evil smelling compounds with these occasions. The abominable stuff which some ladies use makes us shudder to remember as we write. How the habit has arisen or to what it owes its origin we have failed to trace. The compound must be painful in odour and gruesome besides. Many people bring out little cushions, adjust them to their head and neck, and then stretch themselves at full length on the railway carriage, enjoying to the full all the horrors of seasickness that imagination can paint. Once on board the steamer such persons are never happy until the inevitable basin is conspicuously at hand.

### Three Groups of Sea Voyagers.

Now as a matter of experience people for most purposes may be divided into two classes, those who have spirit and those who have none. It is not to be supposed that the entire change of life and habits which a sea trip entails can be commenced with assured comfort or with disregard to ordinary precautions and prudence. The person of spirit at once resolves to take exercise, to eat moderately, and to eat the simplest kinds of food. Those without spirit turn up their toes, and by sitting about do their best to get chilled, and by watching the motions of the vessel to bring on sickness as soon as may be. Between these two classes is a third, those who pride themselves that they are never ill—*i.e.*, sick. This last class suffer untold agonies in their devotion to a theory which has its origin in false pride. Were they content to be wholesomely sick as speedily as possible, with a little trouble, they might bring themselves to be up and about on the second or third day of the voyage, and so secure almost as much enjoyment as the best of sailors. To curl oneself up in a deck chair; to study the Atlantic rollers from such a position; to be content to occupy the attitude and to assume the appearance of a trussed turkey for days together, or, as some do, to assume the shape of an Egyptian mummy, is to abrogate one's manhood, and to prove that the ludicrous aspects of human misery are begot of an absence of pluck, and so to become the just amusement of the observant and the manly.

### At Sea.

Who can picture the miseries of sea sickness, or the joys of the sea to good sailors? To the first an ocean voyage is a misery and a terror, indeed and in truth. To take a promenade on the deck of a steamer in mid-ocean, when a stiff breeze is blowing, is to witness many forms of misery truly, but also to reveal many ludicrous aspects of it too. The deck-chair now so prevalent is a two-fold danger, a trap, and an unmitigated nuisance all in one. The danger is twofold, because it injures (1) those who use it, by perpetuating their sea sickness and misery, and by making them abominably lazy and uncomfortable, the latter feeling being due to an absence of exercise, and to the relinquishment of all effort; and (2) those who follow wiser paths by walking the decks, which are usually so taken up by chairs, that if the sea is at all rough the walkers are liable to bark their shins against them, or to be so hampered for space as to make walking a trouble, if not an impossibility. Yet out of five hundred passengers, quite four hundred are to be seen reclining in chairs from start to finish. These chair characters are very often a sorry crew to look upon: they rarely smile, and are altogether a nuisance to themselves and sometimes even to the well and healthy. They present a woe-begone appearance: all the colours of the rainbow may be seen reproduced in the various hues their skins present, and so the laziness of inaction begets misery, ill-health, and discomfort to the indolent, and makes them, besides, the perfection of comic misery in most cases. Of course, exceptions must, in justice, be made, but, speaking generally, quite ninety per cent. of the chair population would be wiser and better if they could only bring themselves to abandon a practice which makes them permanent subjects of *mal de mer*. It is not easy to describe the joys of the sea to those who care for it. Its wonderful colour, its ever varying surface, its sun sparkling brilliancy, the mountainous and rugged aspect it presents in a storm, the thousand and one rainbows created by the spray, the beauty of the phosphorescence as seen at night, the varying hues, and perfect shape of the waves as they circle and break by the sides of the vessel, its health giving and invigorating properties—all this, and much more, gives the voyager joy indeed. To a busy man seeking entire rest and change the life is often delightful, for during his time at sea he loses all desire to read or to occupy himself, and can eat and sleep to his heart's content. Of course, there are rough days and bad, but even then the beauties of the sea are a constant pleasure to those who can enjoy them. The majority may do this if they will follow the course we have recommended for the adoption of the sensible

Ocean Hotels.

The latest additions to the Atlantic liners are, in fact, huge floating hotels. They will accommodate nearly 600 saloon passengers, or some 1,800 of all grades. They have every kind of appliance, convenience, and luxury to be found in great hotels, and are neither better nor worse than they in most respects. They have also another and a sadder aspect, for the plan on which they are worked creates a system of slavery as vicious and unjustifiable as it is wanton and short-sighted. In one of these big steamers of ten thousand tons or so the number of saloon passengers is so large as to necessitate the duplication of all the chief meals every day. Hence the stewards, or attendants, are worked almost to death from start to finish. The rule is for a steward to be on duty from five a.m. to twelve midnight every day throughout the voyage, and for some days 23 out of the 24 hours when his turn for the night watch comes round. In the same way the cooks are kept over the fires from six a.m. to ten p.m. each day, and even the boys have to work from five a.m. to ten p.m. at night. This is a system of slavery, and nothing else. The wages are small, the work very arduous, and almost incessant from May to November, months both inclusive in each year, and the leave allowed is practically *nil*. If one of these white slaves desires to take a day off he is invariably refused leave, and if he takes French leave he is sure of a wigging, and will probably have to forfeit a day's pay. So tired do these poor young fellows become that they fall asleep over their work, and present the appearance of all having the phthisical habit. It is a fraud upon the saloon passengers that this state of things should be, and all who are wise will avoid travelling by one of these ocean hotels. The fraud consists in taking £35 to £50 from a passenger for a cabin on one of these ships, in alloting to him a white slave so over-worked, and ill-used generally, as to be quite unfit for work, and in leaving the said passenger to make up the inadequate wages to something like a decent sum. What can every right-minded person feel in regard to the capitalists, directors, and owners who permit such evils to prevail on their ships, whilst they receive sometimes in hard cash from £15,000 to £20,000 as the takings of one ocean hotel on a single voyage? What, also, must be thought of the neglect and inefficiency of the Board of Trade, its inspectors and officials, who permit this system of slavery to prevail and flourish without hindrance or redress? Is it to be allowed to continue, having regard to the fact that the hardships of the system are appalling when compared with the admitted evils attaching to the methods of conducting tramway and other businesses, until public opinion stepped in and alleviated them? Yet here we have frightful human misery prevailing on board the "Greyhounds of the Ocean," that is some of the finest steamers afloat, which still present the ludicrous aspect of hundreds of passengers who have paid heavy fares for indifferent treatment, calmly submitting to be deprived of adequate service, and to pay a heavy additional tax in gratuities for it, because the shipowner fails to fulfil his plain duty to the staff he employs, and without whose faithful service his enormous profits could not be earned.

